# Resveratrol biosynthesis, optimization, induction, bio-transformation and bio-degradation in mycoendophytes

**DOI:** 10.3389/fmicb.2022.1010332

**Published:** 2022-10-11

**Authors:** M. A. Abo-Kadoum, Mohamed E. Abouelela, Amal A. Al Mousa, Nageh F. Abo-Dahab, Mohamed A. Mosa, Yosra A. Helmy, Abdallah M. A. Hassane

**Affiliations:** ^1^Department of Botany and Microbiology, Faculty of Science, Al-Azhar University, Assiut, Egypt; ^2^Department of Pharmacognosy, Faculty of Pharmacy, Al-Azhar University, Assiut, Egypt; ^3^Department of Pharmaceutical Sciences, College of Pharmacy, University of Kentucky, Lexington, KY, United States; ^4^Department of Botany and Microbiology, College of Science, King Saud University, Riyadh, Saudi Arabia; ^5^Nanotechnology and Advanced Nano-Materials Laboratory (NANML), Plant Pathology Research Institute, Agricultural Research Center, Giza, Egypt; ^6^Department of Veterinary Science, College of Agriculture, Food and Environment, University of Kentucky, Lexington, KY, United States; ^7^Department of Animal Hygiene, Zoonoses and Animal Ethology, Faculty of Veterinary Medicine, Suez Canal University, Ismailia, Egypt

**Keywords:** resveratrol, endophytic fungi, biosynthesis, key genes, induction, optimization, bio-transformation, bio-degradation

## Abstract

Resveratrol (3,4,5-trihydroxystilbene) is a naturally occurring polyphenolic stilbene compound produced by certain plant species in response to biotic and abiotic factors. Resveratrol has sparked a lot of interest due to its unique structure and approved therapeutic properties for the prevention and treatment of many diseases such as neurological disease, cardiovascular disease, diabetes, inflammation, cancer, and Alzheimer’s disease. Over the last few decades, many studies have focused on the production of resveratrol from various natural sources and the optimization of large-scale production. Endophytic fungi isolated from various types of grapevines and *Polygonum cuspidatum*, the primary plant sources of resveratrol, demonstrated intriguing resveratrol-producing ability. Due to the increasing demand for resveratrol, one active area of research is the use of endophytic fungi and metabolic engineering techniques for resveratrol’s large-scale production. The current review addresses an overview of endophytic fungi as a source for production, as well as biosynthesis pathways and relevant genes incorporated in resveratrol biosynthesis. Various approaches for optimizing resveratrol production from endophytic fungi, as well as their bio-transformation and bio-degradation, are explained in detail.

## Introduction

Endophytes are a group of microorganisms including fungi, bacteria, algae, and actinomycetes that preferentially coexist inside the living plant tissues without causing harmful effects during the life cycle of their host plant (de Bary, 1866; Nair and Padmavathy, 2014). They represent an important partner for the host-plant ecosystem (Aly et al., 2011). It has been reported that they can manipulate host metabolic pathways and participate in the biosynthesis and bioconversion of secondary metabolites that possess biologically active roles in medicine and agriculture (Nisa et al., 2015). Endophytes are the most abundant pool for novel bioactive natural products (Liu et al., 2016a). Fungal endophytes are a specific class of fungi that colonize the intercellular space of host-plant tissues and prompt host growth *via* facilitating nutrient uptake and increasing host resistance against environmental stress (Dwibedi and Saxena, 2018). Endophytic fungi have the metabolic capability to produce secondary metabolites with similar structure and function to their plant host. This is due to co-evolution with their plant host and horizontal gene transfer (HGT) (Aly et al., 2013). Some endophyte biosynthetic enzymes are homologous to those found in plants, corroborating the HGT phenomenon (Aly et al., 2013). Currently, fungal endophytes attracted a lot of attention for their bio-prospecting due to their capacity for the production of novel natural bioactive molecules. These compounds can be used in medicine, agriculture, and industry such as taxol, podophyllotoxin, vincristine, camptothecin, and harzianic acid (Aly et al., 2011; Adeniji, 2019; Dwibedi et al., 2019).

Resveratrol (3, 4, 5-trihydroxystilbene) is a natural polyphenolic compound that belongs to the stilbene family and consists of two benzene rings linked together by isopropyl moiety (Kasiotis et al., 2013). Resveratrol was first isolated from the roots of the poisonous plant *Veratrum grandiflorum* (white hellebore) by Takaoka in 1939 (Dwibedi and Saxena, 2018). Chinese medicine uses resveratrol, which was extracted from *P. cuspidatum* after 20 years, for therapeutic purposes (Nonomura et al., 1963). Resveratrol is widely distributed in numerous plant types such as grapes (*Vitis vinifera*), berries, peanuts (*Arachis hypogaea*), chocolate, tea, and cassia seeds (Burns et al., 2002; Baur and Sinclair, 2006; Wang et al., 2013; Mei et al., 2015). Grapes are considered the most abundant source of resveratrol content compared to other plants (Langcake and Pryce, 1977; Creasy and Coffee, 1988; Aleynova et al., 2021). Resveratrol is produced as phytoalexin to protect plants upon exposure to biotic stress such as fungal and bacterial attacks or abiotic stress such as UV radiation (Adrian et al., 2000; Cichewicz et al., 2000; Bavaresco et al., 2012; Dwibedi and Saxena, 2022). Due to the unique structure and therapeutic properties of resveratrol, it was used for protection against foodborne pathogens, neurological and cardiovascular diseases, diabetes, inflammation, cancer, longevity, free radicals, obesity, and Alzheimer’s diseases (Parker et al., 2005; Aggarwal and Shishodia, 2006; Petrovski et al., 2011; Chen et al., 2013a; Ma et al., 2018). The marvelous role of resveratrol attracted researcher’s attention to maximize their production rate and to find alternative sources for biosynthesis.

**FIGURE 1:**
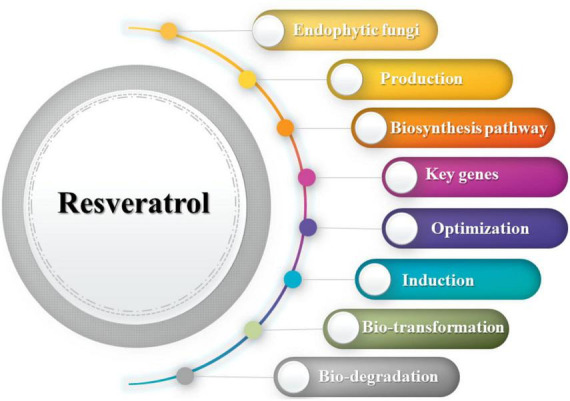
Summary of the review key points.

Extraction of resveratrol from producing plants is a confined process owing to low yields and specific seasons of plant growth (Kiselev, 2011). Mimicking the resveratrol biosynthetic pathway in microorganisms is a promising approach mediated with the aid of using both moving the whole pathway or specific genes to broaden metabolically changed strains, but this approach is steeply priced and time-consuming (Jeandet et al., 2012; Lu et al., 2016). Due to the co-evolution with the host plant, endophytic fungi possess the biosynthetic capacity to produce resveratrol. Bio-prospecting of resveratrol-producing endophytic fungi is a relatively new aspect initiated in the last decade (Dwibedi and Saxena, 2018). In this review, we will emphasize endophytic fungi as a promising alternative source for resveratrol production and underline its biosynthetic mechanism. In addition, we will give highlight on optimization and induction of resveratrol bio-production, bio-transformation, and bio-degradation (Figure 1).

## Resveratrol-producing entophytic fungi

Fungal endophytes with resveratrol production capacity were initially isolated from various parts of their host plant tissues including leaves, stems, roots, and fruits. Powell et al. (1994) isolated resveratrol in high quantity from the grasses, *Festuca versuta* and *F. arundinacea* infected naturally with the endophytic fungus *Acremonium*. The authors found that the obtained resveratrol from endophyte-free plants was reduced and they suggested that this was attributed to the effect of unknown environmental factors, and the reduction of the endophytic fungus *Acremonium* (Powell et al., 1994). Recently, the modulatory effect of endophytic fungi on their host-plant metabolites has been delineated (Nisa et al., 2015).

Several studies have addressed the production of resveratrol from endophytic fungi. Grape plants are the most frequently colonized hosts including *V. vinifera*, *Vitis quinquangularis*, and *Cayratia trifolia* (Roat and Saraf, 2017), together with the Japanese knotweed *P. cuspidatum*. Grapevine is one of the most economically important plants worldwide and a large proportion are directed to wine making (Reynolds, 2017). When the endophytic fungi colonize the grapevine, it increases the nutritional value in berries and prevents the pathogen attack associated with conferring resistance in response to abiotic environmental stress (Aleynova et al., 2021). *P. cuspidatum* is a medicinal plant that contains a high quantity of bioactive secondary metabolites, therefore it was used as traditional Chinese therapy for the treatment of immune diseases, diarrhea, hepatitis, and cancer (Chu et al., 2005). Resveratrol is the main active molecule produced by *P. cuspidatum* (Wang et al., 2013). The incidence of endophytic fungi within *P. cuspidatum* promotes plant growth and enhances the content of bioactive metabolites (Sun et al., 2022).

Resveratrol-producing endophytic fungi are belonging to diverse genera including *Alternaria*, *Botryosphaeria*, *Penicillium*, *Cephalosporium*, *Aspergillus*, *Geotrichum*, *Mucor, Arcopilus, Fusarium, Xylaria*, and *Nigrospora. Alternaria* is the most frequently occurring genus which is isolated from different types of grapevines and *P. cuspidatum* (Shi et al., 2012). Few strains exhibit the ability to produce high and constant levels of resveratrol such as *Alternaria* sp. MG1 (Shi et al., 2012), *Arcopilus aureus* (Dwibedi and Saxena, 2018), *Fusarium equiseti* (Dwibedi and Saxena, 2019a), and *Aspergillus stellifer* (Roat and Saraf, 2020). The rest of the isolated strains showed fluctuated production rates and most of them lost their ability for producing resveratrol, especially during the third sub-culture such as *Cephalosporium, Mucor, Geotrichum*, and *Penicillium* (Shi et al., 2012; Roat and Saraf, 2020). This phenomenon is in accordance with the fact that the fungal capacity to produce active components was reduced over the continuous cultivation for a long time *in vitro* with difficulty to be recovered. The instability of resveratrol production *via* endophytic fungi during *in vitro* culturing discloses the crucial role of the plant host as a partner and reveals that, resveratrol production is a concerted mechanism fine-tuned by host-fungus cross talk. Absence of the plant host results in fluctuated expression of resveratrol-related genes and its metabolic flux in endophytic fungi (Shi et al., 2012; Dwibedi and Saxena, 2019a).

*Alternaria* is a phytopathogenic fungus for many plants such as tomatoes, apples, carrots, and sweet potatoes (Akamatsu et al., 1999; Akamatsu, 2004). *Alternaria* spp. is ubiquities in grapevine, living in a mutual relationship or as a pathogen causing disease through the production of extracellular toxins (González and Tello, 2010). *Alternaria* sp. MG1 strain was isolated from the rachis part of the *V. vinifera* cultivar (Merlot) and showed the highest and most stable resveratrol production rate (104 μg/L) among all isolated strains (Table 1; Shi et al., 2012). Interestingly, the accumulation of resveratrol inside the MG1 strain was initiated at the early stage of the exponential phase contradicting the previous reports in which most of the secondary metabolites are accumulated at the late stage (Chomcheon et al., 2009; Kornsakulkarn et al., 2011). Among thirteen endophytic fungal isolates belonging to *Penicillium*, *Alternaria*, and *Aspergillus* obtained from different parts of the Brinjal plant (*Solanum melongena*), *Alternaria alternata* was the highest resveratrol producer (206 μg/L) (Abdel-Hadi et al., 2022). Whereas, resveratrol is not detected in the Brinjal plant (Singh et al., 2016).

**TABLE 1:** Endophytic fungi produce resveratrol, their host plant, isolation part, used media, incubation time, and the quantity of produced resveratrol according to previous literature.

Endophytic fungi	Host plant	Part	Production media	Incubation time	Resveratrol quantity	References
*Aspergillus stellifer* AB4	*Vitis vinifera* (Merlot)	Leaf	PDA	6–7 days	288 μg/L	Roat and Saraf, 2020
*Alternaria* sp. AB5		Stem			143 μg/L	
*Cephalosporium* sp. AB6		Fruit skin	GA		134 μg/L	
*Penicillium* sp. AB7		Tendril	PDA		123 μg/L	
*Alternaria* sp. AB8		Fruit			14 μg/L	
*Aspergillus* sp. AB9		Rachis			131 μg/L	
*Penicillium* sp. AB10		Root			16 μg/L	
*Alternaria* sp. CD2	*Cayratia trifolia* (wild *Vitis*)	Stem	NA		126 μg/L	
*Cephalosporium* sp. CD3		Rachis	GA		10 μg/L	
*Penicillium* sp. CD4		Fruit			154 μg/L	
*Penicillium* sp. CD5		Root	PDA		152 μg/L	
*Alternaria* sp. EF3	*Vitis quinquangularis* (Rehd)	Rachis	GA1		143 μg/L	
*Aspergillus* sp. EF4		Root	PDA		145 μg/L	
*Arcopilus aureus*	*Vitis vinifera*	Not recorded	PDB	10 days	89.1 mg/L	Dwibedi and Saxena, 2019b
*Fusarium equiseti*					52.3 mg/L	
*Xylaria psidii*					35.4 mg/L	
*Fusarium solani*					31.3 mg/L	
*Alternaria* sp. MG1	*Vitis vinifera* (Merlot)	Rachis	GA1	7 days	104 μg/L	Shi et al., 2012
*Alternaria* sp. MG2					34 μg/L	
*Alternaria* sp. MG3					23 μg/L	
*Alternaria* sp. MG6		Skin			11 μg/L	
*Alternaria* sp. MG7					12 μg/L	
*Alternaria* sp. MP11			PDA		6 μg/L	
*Alternaria* sp. MP15					95 μg/L	
*Botryosphaeria* sp. YG3	*Vitis quinquangularis* (wild *Vitis*)	Stem	GA1		94 μg/L	
*Penicillium* sp. YP30			PDA		17 μg/L	
*Cephalosporium* sp. YG6		Rachis	GA1		35 μg/L	
*Aspergillus* sp. YG8					38 μg/L	
*Cephalosporium* sp. YP7			PDA		21 μg/L	
*Penicillium* sp. YP2		Skin			24 μg/L	
*Geotrichum* sp. HP7	*Polygonum cuspidatum*	Root			43 μg/L	
*Mucor* sp. HP9					21 μg/L	
*Cephalosporium* sp. HG16			GA1		67 μg/L	
*Cephalosporium* sp. HG5		Stem			11 μg/L	
*Alternaria* sp. HG6					123 μg/L	
*Cephalosporium* sp. HG7					56 μg/L	
*Aspergillus niger*	Wine grape (Cabernet Sauvignon)		PDB	3 days	1.48 mg/L	Liu et al., 2016a
*Penicillium* sp.	*Vitis vinifera* (Merlot)	Stem	CDB	7 days	21.9 μg/ml	Dwibedi and Saxena, 2018
*Nigrospora* sp.		Leaf			25.2 μg/ml	
*Aspergillus* sp.					4.4 μg/ml	
*Alternaria* sp.					24.1 μg/ml	
*Aspergillus* sp.					22.4 μg/ml	
Unidentified					11.9 μg/ml	
*Aspergillus* sp.	*Vitis vinifera* (Wild)	Stem			23.9 μg/ml	
Unidentified					13.2 μg/ml	
*Arcopilus aureus*	*Vitis vinifera* (Muscat)	Leaf			89.1 μg/ml	
*Botryosphaeria* sp.	*Vitis vinifera* (Pinot noir)				37.3 μg/ml	
*Penicillium* sp.					15.3 μg/ml	
*Botryosphaeria* sp.	*Vitis vinifera* (Pinot noir)	Leaf and stem	PDB	10 days	15.3 μg/ml	Dwibedi and Saxena, 2019a
*Botryosphaeria* sp.					37.3 μg/ml	
*Fusarium equiseti*					52.3 μg/ml	
*Aspergillus* sp.	*Vitis vinifera* (Merlot)				22.4 μg/ml	
*Nigrospora* sp.					25.2 μg/ml	
*Penicillium* sp.					21.9 μg/ml	
*Aspergillus* sp.					4.4 μg/ml	
*Alternaria* sp.					24.1 μg/ml	
Unidentified					11.9 μg/ml	
*Aspergillus* sp.	*Vitis vinifera* (Wild)				23.9 μg/ml	
Unidentified					13.2 μg/ml	
*Xylaria* sp.	*Vitis vinifera* (Shiraz)				35.4 μg/ml	
*Fusarium* sp.					31.3 μg/ml	
*Xylaria psidii*	*Vitis vinifera* (Shiraz)	Leaf	PDB	10 days	35.43 μg/ml	Dwibedi et al., 2019
*Alternaria alternata* SK.	Brinjal	Leaf	PDB	8–10 days	206 μg/L	Abdel-Hadi et al., 2022
*Quambalaria cyanescens*	*Vitis vinifera*	Stem	CDB	15 days	40 mg/L	Meshram et al., 2022

Dwibedi and Saxena (2019a) reported 11 endophytic fungi isolated from different cultivars of *V. vinifera* and identified as resveratrol producers (Table 1). *A. aureus* #12VVLPM was the most potent producer (89.1 μg/ml) when it is growing on the Czapek Dox broth medium. The production of resveratrol by *A. aureus* was stable during subculturing compared to other isolates (Dwibedi and Saxena, 2018). Interestingly, the #12VVLPM strain was reported to produce the same resveratrol concentration when it is cultivated on potato dextrose broth (PDB) (Dwibedi and Saxena, 2019b), indicating that resveratrol production by *A. aureus* is medium-independent. Genus *Xylaria* is typically found on leaves and seeds of many woody plants (González and Tello, 2010). The strain *Xylaria psidii* #22(P) VVLPM which was isolated from the leaves of *V. vinifera* (Shiraz) was reported to produce less amount of resveratrol (35.43 μg/ml) compared to *A. aureus* when growing on the same PDB medium (Dwibedi et al., 2019).

The endophytic fungi belonging to the genus *Fusarium* are active producers of diverse secondary metabolites such as naphthoquinones that possess antibacterial and antifungal properties (Kornsakulkarn et al., 2011). *Fusarium* has been frequently reported to be associated with *V. vinifera* (González and Tello, 2010; Dwibedi and Saxena, 2019a). Dwibedi and Saxena identified thirteen strains as resveratrol-producing entophytic fungi isolated from *V. vinifera* (Dwibedi and Saxena, 2019a). *Fusarium* includes two strains, *F. equiseti* from the Pinot noir cultivar and *Fusarium solani* from Shiraz. *F. equiseti* is the most potent producer (52.3 μg/ml) owing to the presence of stilbene synthase (*STS*) as a key gene in the resveratrol production pathway (Table 1; Dwibedi and Saxena, 2019a). Furthermore, *Aspergillus* has been frequently isolated from *V. vinifera* and *V. quinquangularis* (Shi et al., 2012; Roat and Saraf, 2020). *A. stellifer* was isolated from the leaf of *V. vinifera* cultivar Merlot and showed stable resveratrol production capacity (288 μg/L) (Roat and Saraf, 2020). Cabernet Sauvignon is the main cultivar of wine grapes in the Shihezi region, China, where *Aspergillus niger* has been isolated and produced 1.48 mg/L of resveratrol (Table 1; Liu et al., 2016a).

*Quambalaria cyanescens* is an endophytic fungus that colonizes corymbia and eucalyptus. *Q. cyanescens* is an active producer of bioactive metabolites that possess a broad spectrum of antimicrobial and anticancer potential such as quambalarine and quambalarine B, respectively (Meshram et al., 2022). *Q. cyanescens* obtained from the stems of *V. vinifera* was the active producer of resveratrol at 40 mg/L and the produced resveratrol is stable over three passages (Table 1; Meshram et al., 2022).

## Host–fungal interaction

Endophytes are crucial for their host plant life in which they promote their growth *via* increasing nutrient availability and acquisition (Yang et al., 2016). They can equip the host plant against biotic and abiotic stress (Yang et al., 2016). Alteration in the phytochemical response of the host upon exposure to endophytes was recorded in a few previous reports, therefore, evaluating plant secondary metabolites should consider the presence of endophytes (Yang et al., 2016). The potential effect of endophytes on the host metabolites has been supported by various evidences. For instance, the production of Chinese medicine “dragon’s blood” by *Dracaena cochinchinensis* is significantly elicited due to the coexistence of the endophytic fungus *Fusarium proliferatum* (Wang et al., 2011). Additionally, co-cultivation of endophytic fungi *A. alternata* and *Epicoccum nigrum* with the grape cells modulate the anthocyanin content and the activity of phenylalanine ammonia-lyase (PAL) indicating the metabolic alteration in the grape cells upon exposure to endophytic fungi (Yu et al., 2020). Endophytes modulate the chemical profile of their host plant through various mechanisms such as biosynthesis and they also export the metabolites directly to the host *via* eliciting signaling cascades and producing enzymes to dictate the host metabolic pathways (Yang et al., 2016).

Endophytic fungi can be utilized as a metabolic regulator to pinpoint the character and quality of the hosting plant, especially for those who produce organoleptic products, like wine and coffee (Yang et al., 2016). Several studies revealed that fungal endophytes can enhance resveratrol production in grape plants. Yang et al. (2016) reported eight endophytic fungi including *Alternaria* spp., *Chaetomium* sp., *Colletotrichum* sp., *Fusarium* sp., *Gibberella* sp., *Nigrospora* sp., and *Xylaria* sp., that were isolated from grapevines and co-cultivated with grapevines in the field. The amount of resveratrol was greatly improved in grapevines upon co-cultivation in leaves and fruits specifically during the maturity stage. Interestingly, *Chaetomium* sp., *Nigrospora* sp., and *Xylaria* sp. were significantly induced resveratrol production than the rest of the isolated fungi (Yang et al., 2016), suggesting the potential effect of applying endophytic consortium to create different patterns of wine grape chemical profile and the resultant wine. Co-cultivation of tissue culture seedlings with endophytic fungi is similar to their daily growth and it was a potential system to enhance the content of bioactive metabolites (Aleynova et al., 2021). The amount of stilbene and trans-resveratrol was increased in *Vitis amurensis* cells co-cultured with endophytic fungi *Biscogniauxia* sp., *Cladosporium* sp., and *Didymella* spp. The highest concentration of trans-resveratrol (2.9 mg/g) dry weight was detected upon induction of endophytic fungus *Biscogniauxia* sp. The induction of trans-resveratrol by endophytic fungi was accompanied by significant up-regulation of resveratrol biosynthesis relevant genes PAL and STS (Aleynova et al., 2021).

Endophytic fungi of medicinal plants promote the accumulation of bioactive secondary metabolites inside their host (Teimoori-Boghsani et al., 2019). The medicinal plant *Rumex gmelini* Turcz (RGT) is a rich source of resveratrol and other bioactive secondary metabolites. RGT possesses diverse pharmacological activities as anticancer, antioxidant, antifungal, antihypertensive, antitussive, antiasthmatic, and antiviral (Zhang et al., 2008). Endophytic fungus *Aspergillus oryzae* promotes the accumulation of resveratrol in tissue culture seedling of RGT by 3.70-folds compared to the non-treated group. However, the level of polydatin (stilbene constituent in *R. gmelini*) was decreased; this may due to the ability of *A. oryzae* to convert polydatin into resveratrol under optimum conditions (Wang et al., 2007). Correspondingly, co-cultivation of *Ramularia* sp. with RGT tissue culture significantly dampens the resveratrol yield (Ding et al., 2018), suggesting the contradicted effect of RGT endophytic fungi.

Phytopathogenic fungi can promote the resveratrol yield in their host plant. *Botryodiplodia theobromae* LBBT HC6-1 and *Botrytis cinerea* FCBC TN1 have significantly induced the level of trans-resveratrol in peanut callus by 8.92 and 16.35 μg/g, respectively (Yang et al., 2010). A similar effect was found in grapevine upon *B. cinerea* FCBC TN1 infection (Paul et al., 1998). Viniferin diglucosides is a resveratrol dimer that was reported to be accumulated in the early stage of the grape fruits’ development in response to *Botrytis* during the Noble Rot infection (Blanco-Ulate et al., 2015). Furthermore, resveratrol derivative, 2,4,6-Trihydroxyphenanthrene-2-*O*-glucoside, was detected in vine leaves upon *Plasmopara viticola* infection (Triska et al., 2012). The chitin layer of the fungal cell wall is a crucial component that is believed to induce resveratrol upon the fungal infection. The amount of trans-resveratrol induction by chitin was two-thirds compared to that elicited by sterilized fungi in peanut callus (Yang et al., 2010). Chitin and its fragments can elicit cascades of defense signaling and promote the activation of the phytoalexin system in the host plant. Melchior et al. (1991) studied the up-regulation of resveratrol biosynthesis-related proteins STS and PAL in the grapevine cell culture treated with fragments of *B. cinerea* cell wall. Those proteins have chitinase and glucanase activities and participate in the defense system *via* destroying the fungal mass (Yang et al., 2010).

## Biosynthesis pathways and relevant genes

**FIGURE 2:**
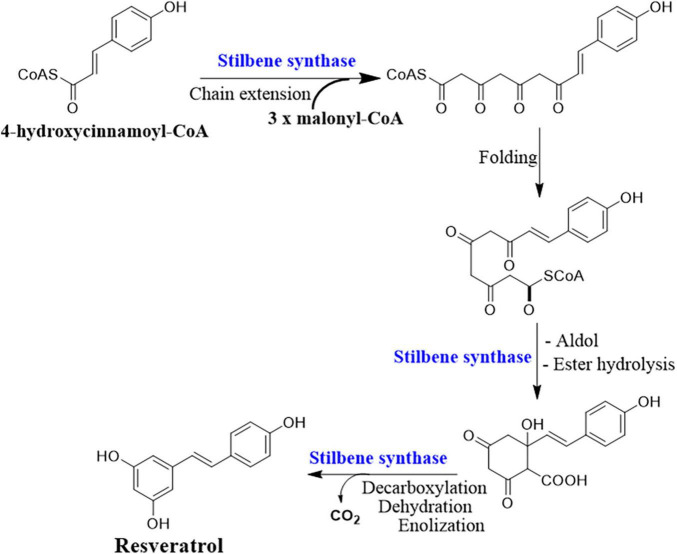
Resveratrol biosynthesis pathway.

Deciphering the resveratrol biosynthesis pathway and its relevant key genes in endophytic fungi is substantial for delicate control of the production condition and overcoming the problems of low and unstable yield (Che et al., 2016b). The complete biosynthetic pathway of resveratrol is only identified in plants as the phenylpropanoid pathway (Figure 2). The key enzymes involved in this pathway are phenylalanine/tyrosine ammonia lyase (PAL/TAL), trans-cinnamate 4-hydroxylase (C4H), 4 coumarate-CoA ligase (4CL), STS, and resveratrol synthase (ST) or chalcone synthase (CHS) (Mei et al., 2015). PAL, C4H, and 4CL are responsible for the biosynthesis of natural phenolic-related metabolites including lignin and flavonols (Mei et al., 2015). PAL and TAL belong to the same protein family with a lyase domain in which they catalyze the breaking of amino acids into α and β-unsaturated acids which are accompanied by removing ammonia (Jendresen et al., 2015). The activity of PAL and TAL is substrate-dependent, in which phenylalanine is specific for PAL and tyrosine for TAL. However, when a point mutation occurred at position 89 (His–Phe), the substrate specificity of TAL switched to phenylalanine (Pinto et al., 2015). The resveratrol biosynthesis pathway is initiated by the deamination of phenylalanine mediated by PAL, and then C4H catalyzes the hydroxylation of trans-cinnamic acid to p-coumaric acid using ATP and coenzyme A. Finally, one 4-coumaroyl-CoA combined with three malonyl-CoA units to form resveratrol is catalyzed by the CHS/ST (Jeandet et al., 2002; Tavares et al., 2013).

**FIGURE 3:**
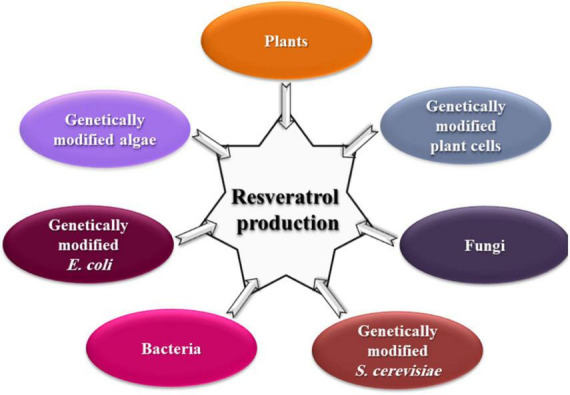
Different natural sources for resveratrol production.

Interestingly, the key enzymes for the resveratrol biosynthesis pathway have been reported separately in some yeast strains. For example, PAL in *Rhodotorula glutinis* and *Rhodosporidium toruloides* (Vannelli et al., 2007a; Wu et al., 2009), TAL in *R. glutinis*, *Trichosporon cutaneum*, and *Saccharothrix espanaensis* (Berner et al., 2006; Vannelli et al., 2007b), 4CL in *Streptomyces coelicolor* (Park et al., 2009), and CHS/ST in *Saccharopolyspora erythraea* (Magdevska et al., 2010). Genetically modified yeast, bacteria, and algae (Figure 3) with resveratrol-producing capacity are successfully constructed either *via* transforming the entire pathway or specific key genes (Liu et al., 2011; Dai et al., 2012; Xiang et al., 2020). However, constructions of key genes originating from plant sources complicated the resveratrol metabolic flux in microorganisms, resulting in low and unstable yield (Che et al., 2016c).

The phenylpropanoid pathway has not yet been identified in microorganisms. However, their key enzymes have been addressed in the endophytic fungus *Alternaria* sp. MG1. The accumulation of intermediate metabolites in *Alternaria* sp. MG1 such as cinnamic acid and p-coumaric acid were detected in association with the activity of upstream enzymes PAL, TAL, C4H, and 4CL in the presence of phenylalanine as a sole substrate, and consistent with the metabolic flux of phenylpropanoid pathway in the plant (Zhang et al., 2013). However, this study failed to detect the downstream enzymes, such as STS, ST, and CHS. Development of molecular techniques such as whole genome sequencing (WGS) and RNA-Seq analysis have helped to understand the complete picture of the resveratrol biosynthesis pathway and related genes in endophytic fungi (Lu et al., 2019). *De novo* transcriptome sequencing of *Alternaria* sp. MG1 was conducted during resveratrol production and a total of 84 genes were annotated encompassing four major resveratrol metabolic pathways that represent 38 coding enzymes (20 for glycolysis, 10 for phenylalanine biosynthesis, 4 for phenylpropanoid biosynthesis, and 4 for stilbenoid biosynthesis). The expression of PAL, 4CL, and CYP73A-related genes was observed (Che et al., 2016b). Additionally, Lu et al. (2019) used the WGS and obtained the same results and found that 4CL and CHS were up-regulated, resulting in a twofold increase in resveratrol production. Resveratrol synthase (ST) was not annotated in the previous studies (Che et al., 2016b; Lu et al., 2019), however, CHS was successfully annotated instead (Jeandet et al., 2010). CHS is an isoenzyme of ST that is responsible for the biosynthesis of naringenin and possesses catalytic activity to form resveratrol, whereas naringenin and resveratrol are produced from the same substrate, p-coumaric acid (Lu et al., 2019). Notably, the amino acid sequences of STSs and CHSs are similar which often hamper their distinction in gene annotation (Yu et al., 2005). Che et al. (2016a) detected the activity of PAL, TAL, C4H, and 4CL in the enzymatic extract of *Alternaria* sp. MG1, confirming the presence of phenylpropanoid biosynthesis pathway. Recently, the functional analysis of CHS and 4CL from *Alternaria* sp. MG1 has been verified. The two genes were heterologously expressed in *Saccharomyces cerevisiae*, and the recombinant strain revealed resveratrol-producing capability reached 113.2 μg/L in the co-culture system (Lu et al., 2021b).

Some key genes are involved indirectly to enhance resveratrol production in *Alternaria* sp. MG1, including uridine-cytidine kinase (UCK) which is responsible for generating uridine monophosphate (UMP) and adenosine diphosphate (ADP) as cofactors participating in resveratrol synthesis, and phosphofructokinase (PFK) which channelizes the metabolic flux to pentose phosphate pathway (Lu et al., 2019). Several cofactors play an important role in resveratrol biosynthesis at different stages. For example, acetyl-CoA is a precursor for the formation of malonyl-CoA, the direct precursor in the last step of resveratrol biosynthesis. The resveratrol flux increased in association with elevated levels of acetyl-CoA (Lu et al., 2019). ATP is a crucial component in the CoA formation by which low ATP yield led to low CoA and subsequently low resveratrol production. Correspondingly, the simultaneous addition of CoA and ATP prompts resveratrol production (Zhang et al., 2013).

## Optimization of resveratrol production

Resveratrol is a natural antioxidant (naturally stilbene occurring phytoalexin) produced by plants in response to resisting the pathogen’s infection and is considered the principal component produced by phytotoxin reaction (Gambini et al., 2015). Resveratrol is a low molecular weight polyphenol compound with high content in grapes, mainly accumulated in high content grape leaves and peels to resist different biotic and abiotic stresses including fungal invasions and exposure to chemicals and UV radiation (Weiskirchen and Weiskirchen, 2016; Hasan and Bae, 2017). Extraction of resveratrol from plants is a classical technique that exhibited remarkable disadvantages, such as prolonged growth time, high cost, reduced yield, and the demand for induction through abiotic and biotic factors (Berman et al., 2017; Wang et al., 2018; Lu et al., 2021a). Chemical synthesis of resveratrol needs ordains complicated steps, utilize hazardous solvents, and produces toxic byproducts (Lu et al., 2019). Therefore, the exploitation of microorganisms to imitate resveratrol production by fermentation is a hopeful strategy (Dwibedi and Saxena, 2018). Endophytic fungi can mimic the productivity of their host phytochemicals due to their association with the host plant (Strobel and Daisy, 2003; Suryanarayanan et al., 2009).

Many researchers have reported the isolation and screening of endophytic fungi from *V. vinifera* for resveratrol production (Dwibedi and Saxena, 2018). Moreover, the isolation of fungal endophytes with the ability to produce resveratrol was affected by the type of isolation media and the plant part from where they were isolated (Shi et al., 2012). In addition, the diversity in endophytic fungal taxa from the same host is due to the incidence of infrequent species in different parts of the plant (Bills and Polishook, 1994). The variation in resveratrol-producing capacity in fungal isolates could be attributed to gene expression fluctuation (Dwibedi and Saxena, 2018). Engineered microorganisms including *S. cerevisiae*, *Escherichia coli*, and *Yarrowia lipolytica* are considered promising candidates for maximizing resveratrol production through diverse fermentation processes (Lu et al., 2016). Due to its scale-up productivity, elevated efficacy, and its cheap price, microbial fermentation including solid state (SSF) and submerged (SmF) has been utilized to produce different valuable economical products (Chemler and Koffas, 2008; Al Mousa et al., 2021; Mohamed et al., 2021a; Hassane et al., 2022a,b).

Extraction and purification of resveratrol produced by SSF or SmF were carried out using various organic solvents. Ethyl acetate is considered the most commonly used solvent which has distinct chemical and biological properties, including extraction of a wide range of bioactive polar and non-polar compounds as well as low toxicity on test strains (Mohamed et al., 2021b). Many novel analytical technologies methods including LC-MS, LC-UVD, and GC–MS was developed to detect resveratrol and its glucoside (Flamini et al., 2013; Fabjanowicz et al., 2018; Grace et al., 2019; Li et al., 2019; Guo et al., 2022). Compared with GC–MS, LC-MS is simpler and more efficient without derivatization (Careri et al., 2004; Grace et al., 2019).

It is well known that the production of secondary metabolites depends on physiological and nutritional parameters (Shi et al., 2012; Liu et al., 2016a; Dwibedi and Saxena, 2019b). The optimization of culture media and fermentation conditions has been critical in the production of metabolites, which is frequently required to improve extraction yield and reaction rate (Chen et al., 2013b). Various physiological and nutritional parameters are important in the fermentative production of bioactive compounds, as well as in developing a process for maximum secondary metabolite production (Shi et al., 2012; Saran et al., 2015). The traditional optimization studies include the variation of one independent factor and maintaining other variables constant, therefore, this design is not competent to indicate the ideal conditions due to its inability to involve interaction among the variables (Tanyildizi et al., 2005; Lau et al., 2020).

The classical approach for process optimization of bioactive compounds is performed by using one variable at a time (OVAT) which is oppressive, monotonous, and time-consuming especially when the number of variables is large; hence, there was a need for an alternative method that is more efficient and less time consuming (Tassoni et al., 2005; Fan et al., 2010). To overcome this problem, another method based on the statistical approach named response surface methodology (RSM) can be used to carry out optimization studies (Saran et al., 2015; Yun et al., 2018). The RSM has been widely used to evaluate and understand the interaction between different critical factors (independent and dependent) affecting the secondary metabolite production by different microorganisms (Bezerra et al., 2008; Silroy et al., 2014), and to investigate the influence of different variables and their interactions at different levels (Priya and Kanmani, 2011) for the development, improvement, and optimization processes through minimizing the number of needed experiments to assess the influence of several variables and their interaction among the independent variables (Yin et al., 2011; Myers et al., 2016). RSM is an integrated statistical and mathematical tool based on the polynomial equation, significance analysis, and stationary point location which can determine the optimum level of variables for the desired response and estimates the interaction between physiological and nutritional parameters (Shi et al., 2012).

Compared with the OVAT method, statistically designed experiments can predict the interaction between the factors in linear and quadratic terms (Lu et al., 2016). Positive diagnostic plots and coefficient of determination (*R*^2^) values show that RSM is very effective at optimizing the parameters (Rajput et al., 2018). Furthermore, the model’s significance was demonstrated using analysis of variance (ANOVA) through Fisher test (F) and low probability (*p*) values, as well as three-dimensional diagrams and contour plots of these parameters using Design Expert^®^ or Minitab^®^ software (Al Mousa et al., 2022a,b; Guo et al., 2022).

Shi et al. (2012) reported that during the cultivation of *Alternaria* sp. MG1 in PDB, biosynthesis of resveratrol started on the first day, reached its maximum production on the seventh day, and then reduced. Consequently, fungal biomass raised to a peak level after 5 days of cultivation. The results revealed that the fluctuation of resveratrol biosynthesis may be attributed to the non-attendance of the host plant during fungal cultivation, which could result in unsteady resveratrol gene expression. Therefore, investigating the key genes encoding the resveratrol pathways would supply basic information about the mechanism of transformation and decreased productivity (Shi et al., 2012). Resveratrol accumulation in *Alternaria* sp. MG1 was found to be directly proportional with increased biomass which suggests that resveratrol could be a constitutive product.

However, the biosynthesis of secondary metabolites by most microorganisms began at the end of the log phase of cell development (Chomcheon et al., 2009; Kornsakulkarn et al., 2011). *Alternaria* sp. MG1 showed preferable resveratrol-producing parameters (422 μg/L) through fermentation in PDB, using Box–Behnken design (BBD) of RSM tactic, where an inoculum size of 6% (spore suspension 1 × 10^4^ spore/ml), a 250-ml flask holding 125 ml medium, 27°C incubation temperature, and shaking at 101 rpm. The influence of the tested optimization parameters on resveratrol accumulation is correlated to MG1 strain growth where decreased inoculum size produced a relatively slow increase in fungal cell development, while excessive inoculum size could cause cell degeneration thus resulting in decreased biomass. Sequentially, a low medium content results in raised inoculum size and vice versa. Therefore, corresponding cell growth results were derived due to the impact of the medium volume and inoculum size. Also, resveratrol production by *Alternaria* sp. MG1 is prospected to be related to its adaptation to the incubation temperature (Shi et al., 2012). The impact of high shaking speed on low resveratrol production could be attributed to the elevated content of oxygen where speed of rotation and oxygen amounts are directly proportional (King et al., 2006; Santos et al., 2011). ANOVA indicated the height significance of the model with *F* = 36.2789 and low *p* = 0.0001, as well as high *R*^2^ = 0.9591 and adjusted *R*^2^ = 0.9327 values (Shi et al., 2012).

Furthermore, Zhang et al. (2013) determined the optimal conditions for resveratrol production in bioconversion using a non-genetically modified *Alternaria* sp. MG1 strain resting cells. The calculated optimal conditions for the BBD and RSM analysis, based on the levels corresponding to the peak of resveratrol production in the single-factor design, were phenylalanine concentration, 4.66 mM; inoculum size, 12.16%; resting time, 21.3 h. The resveratrol production was predicted to be 1.26 μg/L and was 1.38 μg/L in experimental, nearly twofold obtained from basal concentration (0.70 μg/L). ANOVA cleared that the model was significant and well fitted with the data, as explicit from *F* = 8.36, low *p* < 0.01, high *R*^2^ = 0.9149, and adjusted *R*^2^ = 0.8055 values (Zhang et al., 2013).

One variable at a time approach is a conventional method for any optimization study to enhance the production of any biological product. In the present study, this approach was adopted, wherein the different physiological parameters such as temperature, pH, agitation rate, inoculum age, incubation period, and nutritional parameters such as carbon source, and nitrogen source were evaluated and optimized. All experiments were carried out in triplicates and the standard deviation was calculated (Shi et al., 2012; Saran et al., 2015). The optimization of resveratrol production by *A. aureus* #12VVLPM, a strain previously isolated from the Merlot grape (Dwibedi and Saxena, 2018), was performed with help of RSM. Among the variables tested for OVAT, six variables RPM, temp, incubation days, pH, glucose concentration, and peptone concentration were taken for BBD of RSM to assess their impact on resveratrol production by *A. aureus* (Dwibedi et al., 2021).

Optimization results of physicochemical and nutritional parameters using the OVAT approach (in 8 days of incubation, agitation/aeration 120 rpm, temperature 30°C, pH 7.0 at a glucose concentration of 1% and peptone 0.5%) exhibited a 1.23-fold enhancement in production of resveratrol when compared to the initial yield of 89.1 ± 0.08 μg/ml. RSM using BBD resulted in a 1.49-fold enhancement in resveratrol production (133.53 μg/ml) in 8.5 days of incubation, 115 rpm, temperature 30°C, pH 7.0 at a glucose concentration of 1.25%, and peptone 0.625% (Dwibedi et al., 2021). Evaluated ANOVA showed the significance of the model with an *F*-value equal to 21.76 and a probability value less than 0.05. The *R*^2^ value (0.9576) demonstrates that 95.76% variation in the model is caused by three variables and only 4.24% variation could not be explained by the model. The predicted *R*-squared (pred. *R*^2^) of 0.7786 is in reasonable agreement with the adjusted *R*-Squared (adj. *R*^2^) of 0.9136. The adequate precision value measures the signal-to-noise ratio where a ratio of 14.732 greater than 4 is desirable and indicates an adequate signal (Dwibedi et al., 2021).

An enzymatic reaction system, prepared from *Alternaria* sp. MG1 grape endophyte was developed and optimized for bioconversion of resveratrol from glucose. Using the RSM with the enzyme solution, the highest value of resveratrol production (224.40 μg/L) was found under the conditions of pH 6.84, 0.35 g/L glucose, 0.02 mg/L coenzyme A, and 0.02 mg/L ATP within 120 min. The ANOVA for the model elucidated its significance and convenience, as apparent from the *F* = 46.67, low *p* < 0.01, high *R*^2^ = 0.9776, and high adjusted *R*^2^ = 0.9566 values. The lack-of-fit *F* = 1.76 and the accomplice *p*-value of 0.2763 articulated its insignificance (Che et al., 2016a). Enzymatic biosynthesis of resveratrol could be a promising process due to its independence from cell growth, conservative process conditions, and qualification, thus minimizing the inverse impacts facing resveratrol biosynthesis (Park et al., 2010; Yeo et al., 2012).

## Induction of resveratrol production

Resveratrol is a phytoalexin in plants synthesized through the phenylpropanoid pathway in response to a wide range of biotic and abiotic stress factors including fungal infection, UV radiation exposure, anoxic treatment, ozone stress, heavy metals, chemicals, and wounding (Belhadj et al., 2008; Wang et al., 2010; Lu et al., 2016). However, without these stresses, the resveratrol is produced in a low concentration ranging from 0.2 to 16.5 mg/kg fresh weights of existence in leaves, stems, and roots (Wang et al., 2010). The role of resveratrol in fungi is completely ignored; however, it is known that plants produce it as a defense mechanism against fungal attacks. This fact may suggest that resveratrol could exert allelopathic activity against fungal competitors. Volatiles and lysing enzymes are visualized as chemical defenses, which generate several types of stress in such competitors (Chang et al., 2011).

Biosynthesis and extraction of resveratrol from plants ordinarily require months or years for plant development in addition to 2 days or more for the extraction process (Ballard et al., 2010; Chainukool et al., 2014). To shorten the growth period, techniques of plant cell culture (consuming 120 h), genetically modified *S. cerevisiae* (80 h), and genetically modified *E. coli* (20 h) were applied (Watts et al., 2006; Katsuyama et al., 2007; Donnez et al., 2009). Production of resveratrol using genetically modified microorganisms needs 36–96 h and expensive precursors, such as p-coumaric acid, to induce the synthesis of resveratrol in the system (Wang and Yu, 2012). Overexpression of the genes encoding coumarate–CoA ligase and resveratrol synthase, the enzymes involved in the resveratrol synthesis pathway, has also been found to improve resveratrol production by genetically modified yeast and *E. coli* (Beekwilder et al., 2006; Shin et al., 2011; Wang and Yu, 2012). The production of resveratrol by extraction from plants was 3.5–170 mg/L (Ballard et al., 2010; Mantegna et al., 2012), 11–35 mg/L using grape cells cultures (Medina-Bolivar et al., 2007; Kim et al., 2008), 200–315 mg/L by genetically modified plant cells (Kiselev et al., 2007; Dubrovina et al., 2010), 171 and 8.2 mg/L using genetically modified *E. coli* and *S. cerevisiae*, respectively (Watts et al., 2006; Katsuyama et al., 2007; Sun et al., 2015).

In ripe grape fruits, resveratrol biosynthesis is regularly reduced with disruption in the expression of inducible STS, thus revealing increased susceptibility of ripe *Vitis* berries to *B. cinerea* infection (Bais et al., 2000). The activation mechanism of stilbene phytoalexin biosynthesis in grape cells was reported through triggering by *B. cinerea* endopolygalacturonase 1 which can elicit nitric oxide (NO) production *via* NO synthase. NO is involved in the expression of PAL and STS defense genes (Jeandet et al., 2002; Vandelle et al., 2006).

Microbial fermentation using transgenic *Y. lipolytica*, *E. coli*, and *S. cerevisiae*, has advantages including an eco-friendly approach, fast growth rate, simple and inexpensive culture medium, ease of genetic manipulation, environmentally safe and auspicious maximization of resveratrol production (Lu et al., 2016; Wang et al., 2016). However, metabolic engineering *via* inserting of particular genes is a high cost and depletes time. Alternatively, mycondophytes offer considerable potency for biosynthesizing plant-patent secondary metabolites (Venugopalan and Srivastava, 2015). Mycondophytes secured the capacity to produce functional metabolites corresponding to their host plants’ symbionts. Moreover, endophytic fungi exhibited peculiarities of producing high amounts of metabolites that could be toxic, while they own high resistance to their self-produced byproducts (Kusari et al., 2011).

**FIGURE 4:**
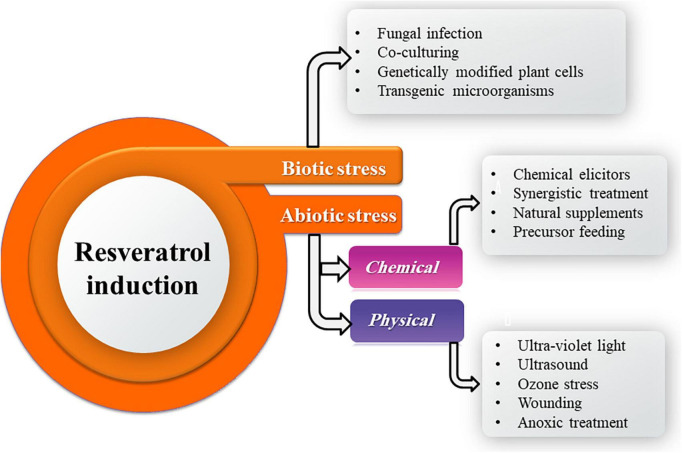
Different elicitors for resveratrol induction.

Resveratrol production by fungi could be perspectively improved by stimulation through induction of chemical, physical, and biological elicitors (Figure 4). Epigenetic modulations in the endophytic fungi ameliorate the spectrum of secondary metabolite production and also conquer a new prospect for regulation of secondary product biosynthesis. Advancements in fungal molecular genetics have revealed that under laboratory culture conditions, many genes of the microbes are unexpressed or phenotypically silent (Dwibedi and Saxena, 2022). Induction of functionality in these genes could be possibly achieved through the *in vitro* use of epigenetic modifiers as well as other interventions such as co-culturing, chemical elicitors, precursors resulting in enhancement of a known host metabolite or novel secondary metabolites that would find applications in pharmaceutical as well as agrochemical industry (Ul-Hassan et al., 2012; Abdulla et al., 2013; Kumar et al., 2016).

For physical induction, UV radiation (350 nm, 20 min) significantly promoted the biosynthesis of resveratrol in *Alternaria* sp. MG1 with highest production (240.57 μg/L) which increased by 45.8% compared with the control at 28°C on the seventh day of incubation. Also, UV irradiation for 20 min significantly upregulated the expression of key genes of the resveratrol biosynthesis pathway by about 3–4 times compared to the control. UV radiation is ordinarily utilized to boost the biosynthesis rate and the catalytic efficiency of fungal enzymes. The increase in the UV treatment time boosts resveratrol production by *Alternaria* sp. MG1, which could be a self-adaptive preservation counter inconvenient environment. Increasing the UV exposure to 30 min, may harm the enzymatic activities and cellular structures, leading to the regression of resveratrol production (Lu et al., 2021a).

On the other hand, ultrasound (40 kHz, 10 min) did not affect *Alternaria* sp. MG1 ability to biosynthesize resveratrol could be ascribed to the insusceptibility of MG1 strain to ultrasound disruption (Lu et al., 2021a). Ultrasound is commonly accounted to affect the growth rates, enzymatic activities, and biosynthesis efficacy of metabolites through enhancing cell permeability across cell membranes (Behzadnia et al., 2020). However, ultrasonic-assisted extraction has the advantages of increasing the extraction rate and reducing the use of organic solvents (Zwingelstein et al., 2020). Ultrasonic cavitation in plants can improve mass transfer in a solid–liquid system, enhance the penetration of medium molecules, accelerate the speed of molecular movement, and quickly penetrate the biomass cells, allowing the extraction solvent to fully contact the biomass, thereby improving the extraction efficiency of target substances and reduce the extraction time (Rodríguez De Luna et al., 2020). In addition, polyphenols are stable and will not be degraded after ultrasonic treatment (Jin et al., 2020). However, the excessive ultrasonic power will degrade or decompose the antioxidant components during the extracted process (O’Brien, 2007).

For chemical induction, *Alternaria* sp. MG1 resveratrol biosynthetic ability was positively influenced by ethanol which acts as a carbon source and increased resveratrol accumulation by 26.31% in a concentration-dependent (Lu et al., 2019). Lu et al. (2021a) found that sodium butyrate at a concentration of 100 μm exerted a negative impact against *Alternaria* sp. MG1 resveratrol production capability and attributed that to the fungal growth reduction which causes deficiency in supplements representing essential precursors for resveratrol biosynthesis. Moreover, Mohammadipanah et al. (2020) speculated that the induction ability of sodium butyrate could be concentration-dependent. On the other hand, El-Hawary et al. (2018) and Mohammadipanah et al. (2020) reported a histone deacetylases inhibition ability of sodium butyrate, which permits the expression of many silent genes. Consequentially, several enzymes are activated, and new secondary products are biosynthesized due to the enhancement role of sodium butyrate. Oligomeric proanthocyanidin (OPC), an important nutrient component in grape fruit, at 100 μm had a positive influence on resveratrol production (255.01 μg/L) in *Alternaria* sp. MG1, which represents an enhancement of 60.63% over the control.

Meanwhile, resveratrol biosynthesis was prohibited at 200 μm OPC which exhibited the impact of OPC on resveratrol cumulation was concentration-dependent. Lu et al. (2021b) revealed the harmony of the expression of the key genes in the resveratrol pathway and resveratrol production after OPC treatment. OPC activates Ca^2+^ channels *via* interacting with Ca^2+^ signal-related proteins, and calcium-dependent protein kinases (CDPKs), thereby activating the upregulation of downstream resveratrol and related genes to resist the stress environment caused by OPC.

Villa-Ruano et al. (2021) found that variable concentrations of amino acid precursors phenylalanine and tyrosine (50–500 mg/L) stimulated resveratrol production by *A. aureus* MaC7A from 127.9 to 221.8 mg/L in basic cultures developed in PDB (pH 7) added with 10 g/L peptones at 30°C. While, among the tested volatiles monoterpenes (limonene, camphor, citral, thymol, and menthol), citral (50 mg/L) enhanced resveratrol production until 187.8 mg/L in basic cultures. Moreover, mixtures of hydrolytic enzymes (Glucanex 100 mg/L), as elicitors, boosted fungal resveratrol productivity (198.3 mg/L). Optimized batch cultures containing tyrosine, citral, thymol, and Glucanex (200, 50, 50, and 100 mg/L, respectively) produced resveratrol up to 237.6 mg/L. Thus, suggests an increase in the production of resveratrol by *A. aureus* MaC7A at mixtures of isoenzymes and low concentrations of volatiles. Endophytic fungus *A. aureus* #12VVLMP, isolated from leaves of *V. vinifera* – Merlot variety and produced 89.13 μg/ml resveratrol extracellularly as a basal concentration (Dwibedi and Saxena, 2018), was inoculated into 50 ml of pre-sterilized PDB was amended with 500 μl of crude methanolic extracts of grape seed and grape skin, as natural supplements, with estimated resveratrol concentrations of 17.3 and 3.3 μg/ml in these extracts, respectively, and incubated at 26 ± 2°C, 120 rpm for 7 days.

Salicylic acid and cyclodextrin (1% v/v), chemical elicitors, were used as chemical elicitors used in the liquid culture of *A. aureus* for enhancing resveratrol production. p-Coumaric acid and cinnamic acid, as precursor feeding, were evaluated as precursors for resveratrol biosynthesis at 1% v/v in 50 ml PDB. An increase of 27.7% was recorded over the basal concentration of 89.13 μg/ml in the case of grape seed extract, while only 13.7% enhancement was observed in the case of grape skin extract. A significant reduction in the total resveratrol content was observed with salicylic acid and cyclodextrin treatment. The production of resveratrol by the use of p-coumaric acid was 1.35% (90.33 μg/ml) higher than the basal concentration produced by *A. aureus*, while a 0.27% increase was observed in the case of cinnamic acid (Dwibedi and Saxena, 2022). The synergistic impact of several elicitors is an efficient trend to raise the biosynthesis of secondary products obtained from microbial or plant origins. Synergistic treatment of UV irradiation (350 nm, 20 min) and OPC (100 μm) increased the production of resveratrol in *Alternaria* sp. MG1by 70.37% (276.31 μg/L) compared to control (Lu et al., 2021b).

Epigenetics permits targeting a wide variety of fungi without any prior information about their genome. Epigenetic modifiers can therefore be used as a strategy to regulate gene transcription in endophytic fungi for modulating gene expression and inducing the biosynthesis of desired secondary metabolites (Dwibedi et al., 2019). The endophytic *X. psidii* #22(P) VVLPM derived from the leaf of *V. vinifera* leaves showed enhanced resveratrol concentrations, in comparison with control (35.43 μg/ml), treatment with 5 μm suberoylanilide hydroxamic acid (SAHA) produced 52.32 μg/ml resveratrol and with 10 μm 5-azacytidine (AZA) (48.94 μg/ml) pursued by 10 μm SAHA (41.10 μg/ml), and 5 μm AZA (37.72 μg/ml) that acting as chemical elicitors and epigenetic modulators (Dwibedi et al., 2019). Chemical enhancers have been used to target biosynthetic pathways, where AZA induces hypomethylation of the stocktickerDNA (Cherblanc et al., 2012, 2013), while SAHA acts as a histone deacetylase inhibitor (Williams et al., 2008; Sharma et al., 2017). Thus, they exemplify a spectacular chemical implementation for the expression of cryptic genes which are not expressed under standard laboratory conditions (Dwibedi and Saxena, 2019a).

For biological induction, co-culturing of endophytes *Alternaria* sp. MG1 (CCTCC M 2011348) and *Phomopsis* sp. XP-8 (CCTCC M 209291) showed an evident influence on resveratrol biosynthesis, which raised its accumulation by 40%, and altered the patterns of secondary metabolites in MG1 by enhancing stilbenes biosynthesis and inhibiting the synthesis of lignin compounds and originating new flavonoids [(+)-catechin, naringin, and (±)-taxifolin]. Accordingly, the microbial interaction may induce the expression of related cryptic gene clusters (Lu et al., 2021b). Whereas co-cultivation of *A. aureus* #12VVLMP with *Fusarium* sp. #19 VVLPM enhanced the resveratrol production by 9.4% (97.47 μg/ml) over 89.13 μg/ml resveratrol extracellular production by *A. aureus* as a basal concentration (Dwibedi and Saxena, 2022).

Several genes involved in the biosynthesis of secondary metabolites remain silent under optimal laboratory conditions and are known to be induced by epigenetic regulation of microbial endophytes (Rutledge and Challis, 2015; Fischer et al., 2016). Bioactive natural compounds can modify histones and DNA methylation and histone epigenetically which results in cryptic gene activation (Meeran et al., 2010). Resveratrol is well known to be potent to introduce epigenetic modification requested to induce silent gene expression. The treatment of *Colletotrichum gloeosporioides* by 10 mg/ml of crude methanolic extract of grape skin (resveratrol enriched natural supplements), for 21 days at 26°C, promoted the production of several cryptic components that were not detected in the control samples. The analytical profile of the secondary metabolites in the treated culture showed the existence of 37 compounds, while the untreated control culture displayed 34 compounds. Twenty cryptic compounds were detected within *C. gloeosporioides* cultures supplemented with grape skin extract. Seventeen compounds were detected in both the treated and control cultures, while certain metabolites were missed in the treated culture (Sharma et al., 2017).

## Resveratrol bio-transformation and bio-degradation

Because of their biological importance, the production of resveratrol as well as its derivatives through bioconversion and biotransformation has been the subject of intensive research studies. Resveratrol is a biodegradable compound that can be metabolized into various bioactive analogues *via* microorganisms. Incubating resveratrol with the cell filtrate of *Bacillus cereus* results in the production of resveratrol 3-O-β-D-glucoside (piceid) as an active analog (Cichewicz and Kouzi, 1998). Similarly, *Streptomyces* sp. isolated from *P. cuspidatum* showed the ability to transform resveratrol into its methylated form 3, 5, 4′-trimethoxy-trans-stilbenes. Interestingly, the transformed derivative exerted stronger anticancer activity in a concentration-independent manner compared with the parent compound (Jiewei et al., 2018).

A number of endophytic fungi isolated from selected plants were analyzed for their resveratrol-producing ability. Few studies have addressed the biotransformation of resveratrol by mycoendophytes. The endophytic fungus *Penicillium* sp. derived from *P*. *cuspidatum* can transform resveratrol when cultured in a PDB medium into trans-3,5-dimethoxy-4′ hydroxystilbene which is known as pterostilbene. The latter is a dimethylated derivative of resveratrol; this modification improves the biological efficacy, membrane permeability, and metabolic stability of pterostilbene over resveratrol. Hence, pterostilbene is considered the next generation of resveratrol and is expected to play a crucial pharmacological role in several human diseases (Xu et al., 2020). Resveratrol is produced by peanuts and accumulated in the surrounding soil conferring allelopathic impact in the peanut mono-cropping systems. This phenomenon undermines the peanut output through different strategies such as inhibiting nodule formation, decreasing the microbial abundance in the soil, and reducing the level of soil carbon. Endophytic fungus *Phomopsis liquidambari* transform approximately 97% of resveratrol *in vitro* and under soil conditions into 3,5-dihydroxybenzaldehyde and 4-hydroxybenzaldehyde, and further oxidize them to 3,5-dihydroxybenzoic acid and 4-hydroxybenzoic acid. The activity of *P*. *liquidambari* toward resveratrol is attributed to the enhanced expression of resveratrol cleavage oxygenase (*resB3)* and other related genes upon resveratrol induction (Wang et al., 2019). Incubation of resveratrol with resveratrol cleavage oxygenase (Rco1) derived from *Ustilago maydis* results in cleaves of Ca–Cb interphenyl double bond, Rco1 is the first eukaryotic enzyme for resveratrol biodegradation (Brefort et al., 2011).

Various strains of the plant pathogen *B. cinerea* demonstrated the ability to degrade resveratrol accompanied by pathogenicity to grapevines *Vitis rupestris*. The degradation was related to the presence of laccase activity in the culture filtrates contradicted the above study. Interestingly, laccase can be produced in high amounts in a culture medium lacking resveratrol as an inducer (Sbaghi et al., 1996). Resveratrol accumulates in grape plants in response to grapevine trunk diseases (GTD). *Neofusicoccum parvum* and *Diplodia seriata* are two major fungi associated with GTD, these fungi are able efficiently to metabolize resveratrol. Higher diversity of resveratrol metabolization products was found with enzymes of *N. parvum* compared to *D. seriata* (Labois et al., 2021).

The investigation of *Alternaria* sp. MG1 cultured on liquid potato dextrose culture resulted in the production of resveratrol on the first day and reached its peak levels on day 7 (Shi et al., 2012). Moreover, the filamentous fungi *A. aureus* MaC7A was able to produce resveratrol under controlled conditions of amino acid precursors (PHE and TYR), and low monoterpenes (limonene, camphor, citral, thymol, and menthol) percentile, and mixtures of hydrolytic enzymes (Glucanex) as elicitors for boosting fungal resveratrol. The peak levels of resveratrol and biomass were maintained during days 6–8 and decreased from days 10–12 without any loss of biomass (Villa-Ruano et al., 2021). In addition, *A. niger* showed stable genetic properties producing a high percentage of resveratrol (Liu et al., 2016b). *Q. cyanescens*, a fungal endophyte of *V. vinifera*, has shown a high amount of resveratrol production (Meshram et al., 2022). *A. stellifer* AB4 isolated from the leaves of *V. vinifera* has shown stable production capability of resveratrol with peak production on day 9 of fermentation (Roat and Saraf, 2020).

**FIGURE 5:**
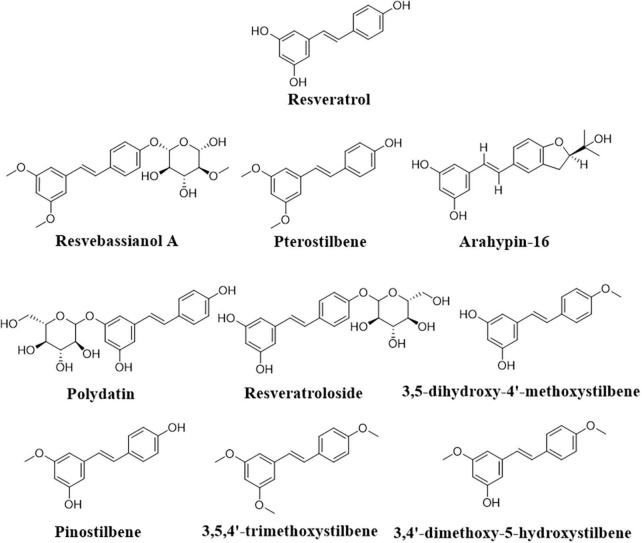
Representative structure of resveratrol biotransformed derivatives.

Further, the transformation of resveratrol into pterostilbene (Figure 5) to increase the stability and bioefficiency was achieved by endophytic *Penicillium* sp. JQ228238 and *Penicillium* sp. F5 from *Polygonum cuspidatum* (Xu et al., 2020; Liu et al., 2021). Moreover, arahypin-16, a biotransformed resveratrol derivative, has been isolated from whole-cell fermentation of *Aspergillus* sp. SCSIOW2 (Wang et al., 2016). The fungus *Beauveria bassiana*, one of the most frequently used microorganisms for the biotransformation of polyphenols, showed the ability of resveratrol conversion to resvebassianol A, a glycosylated metabolite of resveratrol (Darlami and Shin, 2021).

Many investigations into optimizing the bioconversion of polydatin to resveratrol have been conducted. *A. niger* and yeast were able to biotransform polydatin to resveratrol in *P. cuspidatum* roots (Wang et al., 2016). *Dekkera bruxellensis* demonstrated promising activity in hydrolyzing glycosidic-linked resveratrol from *P. cuspidatum*, which could significantly increase resveratrol production (Kuo et al., 2017).

On the other hand, the enzymes obtained from different organisms showed a potential ability for resveratrol biotransformation. The unspecific peroxygenases obtained from the basidiomycetes *Agrocybe aegerita*, *Coprinopsis cinerea*, and *Marasmius rotula* were able to catalyze the hydroxylation of the pin to resveratrol (Aranda et al., 2018). Furthermore, an immobilized enzyme derived from the endophytic fungus *Alternaria* sp. MG1 was successful in the bioconversion of glucose to resveratrol, maintaining high resveratrol production during recycling use two to five times within 2 h for each cycle (Zhang et al., 2013; Che et al., 2016a). The cyclodextrin glycosyltransferase (CGTase) produced by *Paenibacillus* sp. RB01 was used to catalyze the transglycosylation reaction from glycosyl donors (starch, β-cyclodextrin, or maltoheptaose) to resveratrol, resulting in resveratrol glycoside formation (Anurutphan and Prousoontorn, 2014).

According to Schouten et al. (2002), resveratrol is converted to a mycotoxic compound by a specific laccase of *B. cinerea*, which causes autotoxicity by catalyzing the oxidation of phenolic compounds and the reduction of molecular oxygen into water and may be involved in *V. vinifera* grape resistance to *B. cinerea* infection. Recently, genetic bioengineering has played an important role in resveratrol bioproduction. The ability of *E. coli* and *S. cerevisiae* engineered to express the resveratrol *O*-methyltransferase gene from *V. vinifera* to produce pterostilbene from resveratrol was confirmed (Wang et al., 2015). On the other hand, Camacho-Zaragoza et al. (2016) adapted a co-culture system comprised of two populations of engineered *E. coli* strains with genes to produce malonyl-CoA and provide it to the STS for yielding resveratrol. The co-culture of these strains produced 22.58 mg/L resveratrol from 10 g/L glycerol after 30 h while keeping the growth rates of both strains during co-culture cultivation (Camacho-Zaragoza et al., 2016). A similar co-culture design was used for the biosynthesis of polydatin and resveratrol side glycosylated resveratrol products (Figure 5), as well as the conversion of exogenous *p*-coumaric acid *via* over-expression of 4-coumarate-coenzyme A ligase and STS, and to boost UPD-glucose formation (Chen et al., 2019).

In an *E. coli* system containing codon-optimized *O*-methyltransferase genes from sorghum in addition to the resveratrol biosynthetic genes, an artificial biosynthetic pathway was able to produce methylated resveratrol derivatives, such as 3,5-dihydroxy-4′-methoxystilbene, pinostilbene, 3,4′-dimethoxy-5-hydroxystilbene, and 3,5,4′-trimethoxystilbene (Figure 5; Kang et al., 2014). The *Bacillus aryabhattai* endophytic bacteria isolated from the rhizome tissue of *Reynoutria japonica* as well as *Bacillus safensis* has also shown potential transformation of polydatin to resveratrol (Hu et al., 2019; Liu et al., 2020).

## Conclusion

It has been reported that several plant species can produce pharmacologically significant natural products that could be used as a scaffold for the development of new therapeutic candidates. Despite their importance, some of these compounds are obtained in small quantities from plants and cannot be synthesized on a large scale. Fungal endophytes have recently emerged as valuable sources of bio-effective metabolites, either through the production of the same product as host sources or through biotransformation to achieve large-scale production of targeted compounds. Since the stilbene compounds resveratrol and its derivatives have been shown to have important bioactive roles in medicine, we reported here that advancements in fermentation process optimization and genetic manipulation technologies play a critical role in supplying a maximum fungal production of these bioactive metabolites with the least amount of effort and cost. Furthermore, tracking the biosynthesis pathway and key genes involved in biotransformation paves the way for it to be enhanced and improved have been reported. As a result, studying mycoendophytes in various plant species can provide an amazing avenue for maximizing resveratrol production and supplying a consistent yield, as it is an important candidate against various human diseases.

## Author contributions

All authors listed have made a substantial, direct, and intellectual contribution to the work, and approved it for publication.
